# Change in DNA Methylation Patterns of *SLC6A4* Gene in the Gastric Mucosa in Functional Dyspepsia

**DOI:** 10.1371/journal.pone.0105565

**Published:** 2014-08-22

**Authors:** Tomomitsu Tahara, Tomoyuki Shibata, Masaaki Okubo, Kazuya Sumi, Takamitsu Ishizuka, Masakatsu Nakamura, Mitsuo Nagasaka, Yoshihito Nakagawa, Naoki Ohmiya, Tomiyasu Arisawa, Ichiro Hirata

**Affiliations:** 1 Department of Gastroenterology, Fujita Health University School of Medicine, Toyoake, Aichi, Japan; 2 Department of Gastroenterology, Kanazawa Medical University, Uchinadamachi, Ishikawa, Japan; University of Navarra, Spain

## Abstract

**Background:**

The neurochemical serotonin (5-HT) is an important signaling molecule in the gastrointestinal motor and sensory functions. A key regulator of 5-HT levels is the transmembrane serotonin transporter (5-HTT; SLC6A4) that governs the reuptake of 5-HT. Recent studies have indicated 5-HTT expression may be regulated by epigenetic mechanisms. We investigated DNA methylation status of *SLC6A4* gene in the gastric mucosa from functional dyspepsia (FD) because of their potential role in dyspeptic symptoms.

**Methods:**

Endoscopic gastric biopsies were obtained from 78 subjects with no upper abdominal symptoms and 79 patients with FD. Bisulfite Pyrosequencing was carried out to determine the methylation status of promoter CpG islands (PCGIs), promoter non-CpG islands (PNCGIs) and gene body non-CpG islands (NPNCGIs) in the *SLC6A4* gene. Gene expression was examined by real-time PCR.

**Results:**

In overall, methylation level of PCGIs was significantly lower in FD compared to control subjects (*p* = 0.04). On the other hand, methylation level of NPNCGIs was significantly higher in FD compared to control subjects (*p* = 0.03). Lower methylation level in PNCGIs was highlighted in the patients with PDS (*p* = 0.01), while higher methylation level in NPNCGIs was more prominent in the patients with EPS (*p* = 0.017). Methylation levels of PCGIs and PNCGIs were inversely correlated, while methylation levels of NPNCGIs was positively correlated with SLC6A4 mRNA levels in FD patients.

**Conclusions:**

Our data suggest that change in DNA methylation pattern of SLC6A4 in the gastric mucosa may have a role for developing FD. A role of epigenetics for developing FD needs to be further evaluated.

## Introduction

Functional dyspepsia (FD) is clearly the commonest cause of dyspeptic symptoms in the West and increasingly in other parts of the world [1], affecting about 25% of the population [2]. The latest definition of this includes the presence of “chronic or recurrent symptoms centered in the upper abdomen in the absence of any organic, systemic or metabolic disease that is likely to explain the symptoms” [3]. FD is a heterogeneous disorder, which does not have a well-established pathophysiology. Gastrointestinal motor abnormalities [4], [5], altered visceral sensation [6], [7], psychosocial factors [8], [9], genetic factors [10] have all been suggested as pathophysiologic mechanisms but the result is not conclusive.

Serotonin (5-hydroxytryptamin, 5-HT) is an important signaling molecule affecting gastrointestinal motor and sensory functions. Previous reports have suggested the involvement of 5-HT and reduced level of mucosal serotonin in functional gastrointestinal disorders [11]. The serotonin transporter protein (SERT) or 5-HT transporter (5-HTT) encoded by *SLC6A4* gene maps on chromosome 17q11, mainly responsible for the reuptake of serotonin into mucosal epithelial cells and enteric neurons [12]. SERT is a significant neurotransmitter and paracrine signaling molecule in the gut [13]. SERT signaling plays an important role in the modulation of brain-gut communication and functional gastrointestinal disorders [13]. Several reports have demonstrated that genetic polymorphism in *SLC6A4*, affecting 5-HTT expression level is associated with susceptibility to functional gastrointestinal disorders [14]–[16]. Other than genetic polymorphism, 5-HTT expression may also be regulated by epigenetic mechanisms. The methylation status of the *SLC6A4* promoter was suggested to play a role in governing SLC6A4 mRNA levels [17]. Change in SLC6A4 methylation level has been associated with psychiatric disorders, such as depression [18].

Because of their potential role in functional dyspepsia current study aimed to determine the DNA methylation status of *SLC6A4* gene in the gastric mucosa in FD. To obtain detailed information about methylation status, we evaluated the methylation levels of *SLC6A4* across the gene, including promoter CpG island (PCGI), promoter non-CpG island (PNCGI), and gene body non-promoter non-CpG island (NPNCGI). Our result characterized change in methylation status in gastric mucosa in FD, supporting the role of epigenetic disturbance as the pathophysiologic mechanisms of FD.

## Methods

### Ethics statement

This study was approved by the Human Research Ethics Committee of the Fujita Health University School of Medicine. Each participant provided a written informed consent for the clinical and laboratory data to be used and published for research purposes. The study was conducted according to the principles expressed in the Declaration of Helsinki.

### FD patients and controls

Enrolled were 79 FD patients and 78 asymptomatic subjects (control) attending the endoscopy center of Fujita Health University from January 2005 to April 2009. Based on the Rome III criteria, FD patients were defined, as having a primary complaint of at least 3 months of either continuous or intermittent dyspepsia, onset at least 6 months before, predominantly located in the upper abdomen irrespective of using H2-receptor antagonists or proton-pump inhibitors. Among them, 43 and 24 FD was diagnosed as epigastric pain syndrome (EPS) and postprandial distress syndrome (PDS), respectively, while 12 were diagnosed as both EPS and PDS. The definition of asymptomatic subjects (control) was negative for dyspeptic symptom within last 12 months. We confirmed that control subjects did not include subjects who had received proton-pump inhibitory drugs or H2-receptor antagonists within last 12 months. All subjects performed upper gastroscopy to verify there are no significant upper gastrointestinal findings such as peptic ulcer disease, reflex esophagitis, and malignancies. Face-to-face history and physical examination including blood test, abdominal ultrasonography, and electrocardiography were also conducted to rule out any organic, systemic or metabolic disease that is likely to explain the symptoms.

### Detection of H. pylori Infection

The *H. pylori* infection status was determined on the basis of histology using biopsies from uninvolved mucosa from greater curvature of gastric antrum and corpus, serology measuring anti-*H. pylori* antibody titer by enzyme immunoassay (SRL, Tokyo, Japan) and the 13C-urea breath test (UBIT, Otsuka, Tokyo, Japan). Infection was diagnosed when at least one of these tests was positive. We confirmed that the study participants did not include subjects who had past treatment of *H. pylori* eradication.

### Gastric sample collection

During upper gastroscopy, biopsy specimens were taken from greater curvature of the lower gastric body. The specimens were immediately frozen and stored at −80 degree until use. Genomic DNA was extracted using standard protein precipitation method. For 38 FD patients, RNA was extracted using Trizol (Invitrogen, Carlsbad, CA).

### Methylation analysis of SLC6A4

**Figure 1:**
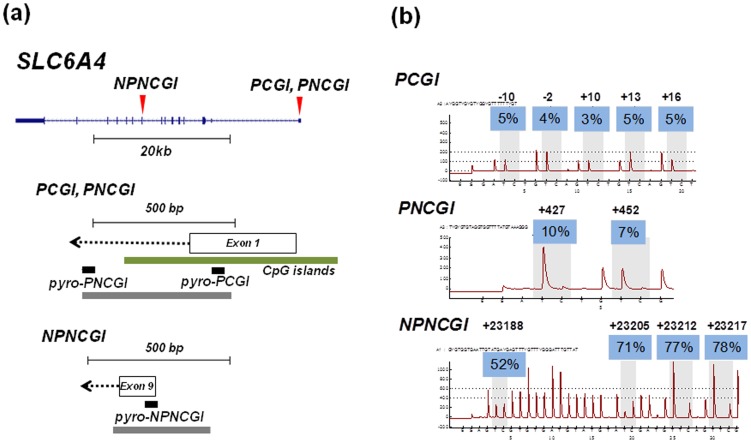
Structure of *SLC6A4* gene and the PCGI, PNCGI and NPNCGI regions analyzed by bisulfite pyrosequencing (black bars) and bisulfite cloning sequencing (grey bars). CpG islands represent green bar (a). A representative pyrogram from a clinical sample (b).

Bisulfite modification was carried out using 500 ng genomic DNA using the EZ DNA Methylation-Gold Kit (D5007, Zymo Research, Orange, CA, USA) according to the manufacturer's instructions. Methylation status of *SLC6A4* gene was investigated by bisulfite pyrosequencing, highly quantitative and reproducible method (Fig. S1) and bisulfite cloning sequencing. We consulted Blat - UCSC Genome Browser (https://genome.ucsc.edu/FAQ/FAQblat.html) to get RefSeq summary of human *SLC6A4* gene (NM_001045). Detailed annotation of promoter, non-promoter, CpG island, non-CpG island and exon-introns of *SLC6A4* gene was obtained. We examined mehylation status of *SLC6A4* gene promoter CpG island (PCGI: positions −10, −2, +10, +13 and +16), which has been reported to have influence on serotonin transporter mRNA levels [17]. To obtain detailed information about methylation status across *SLC6A4* gene, we also evaluated the methylation levels in the promoter non-CpG island (PNCGI: positions +427, +452), which besides to PCGI. Moreover, we searched down stream of *SLC6A4* gene to find CpG sites within the non-promoter gene body legion. We found CpG sites in the exon 9 of the *SLC6A4* gene (positions +23188, +23205, +23212, +23217) and then examined these CpG sites as the gene body non-promoter non-CpG island (NPNCGI) (Fig. 1).

Bisulfite pyrosequencing was carried out using PSQ24 system with Pyro-Gold reagent Kit (QIAGEN, Tokyo, Japan), and the results were analyzed using PyroMark Q24 software (QIAGEN). The primers used for pyrosequencing are listed in Table S1. For bisulfite sequencing, amplified PCR products were cloned into pCR^TM^4-TOPO vector (Invitrogen), and at least 10 clones from each sample were sequenced using an ABI3730XL automated sequencer (Applied Biosystems, Carlsbad, CA, USA). Primer sequences and PCR product sizes are listed in Table S2.

### Gene expression analysis of SLC6A4

Two micrograms of total RNA were reverse-transcribed using High Capacity cDNA Reverse Transcription Kit (Applied Biosystems). cDNA was confirmed by amplifying glyceraldehyde-3-phosphate dehydrogenase (GAPDH). Primer sequences and PCR product sizes are shown in Table S3. Quantitative reverse transcription-PCR (RT-PCR) was carried out using the iTaq SYBR Green Supermix (Bio-Rad, Hercules, CA, USA) and 7900HT Fast Real-Time PCR System (Applied Biosystems). SDS software (Applied Biosystems) was used for comparative ΔCt analysis.

### Statistical analysis

Methylation and gene expression levels of two groups were compared using the Student's t-Test. The correlation between methylation levels of two groups were assessed using the Spearman correlation analysis. p value<0.05 was considered statistically significant.

## Results

### Study population and methylation status of SLC6A4 in the gastric mucosa

Clinicopathological features of subjects are shown in Table 1. There was no significant difference regarding age, gender, and *H. pylori* positivity among FD and control groups.

**Table 1:** Clinicopathological features of subjects.

Variables	FD*	Control	*p* value
Total number	79	78	-
Age (mean+/−SE)	57.0+/−17	59.8+/−1.6	0.23
Male (%)	37(46.8%)	45(57.7%)	0.2
*H. pylori* positive subjects (%)	32(40.5%)	40 (51.2%)	0.2

*: FD consist of 43 epigastric pain syndrome (EPS), 24 postprandial distress syndrome (PDS), and 12 EPS/PDS according to Rome III criteria.

**Figure 2:**
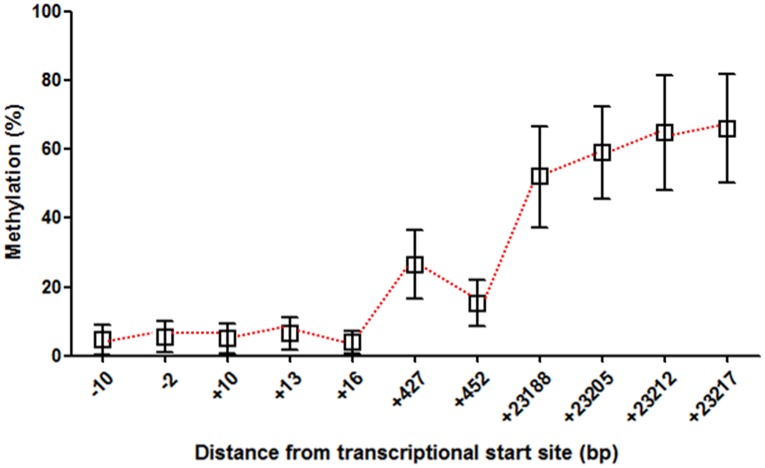
Methylation status of *SLC6A4* gene in the gastric mucosa in relation to the distance from transcriptional start site. Average methylation of each sites analyzed were expressed as mean +/−SEM.

Methylation status of PCGI (positions −10, −2, +10, +13 and +16), PNCGI (positions +427, +452), and NPNCGI (positions +23188, +23205, +23212, +23217) were determined by bisulfite pyrosequencing (Fig. 1, Fig. S1). Fig. 2 shows averaged methylation levels of *SLC6A4* I in all sites. *SLC6A4* methylation level in the gastric mucosa was lowest in PCGIs (5.1+/− 0.29%) and highest in NPNCGIs (60.4+/−1.1%), while PNCGIs presented intermediate methylation level (20.9+/−0.64%).

**Figure 3:**
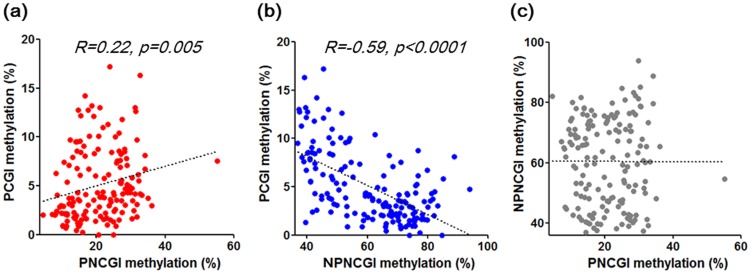
Correlation of *SLC6A4* methylation status among PCGI, PNCGI and NPNCGI. Avarage methylation levels of PCGI (positions −10, −2, +10, +13 and +16), PNCGI (positions +427, +452) and NPNCGI (positions +23188, +23205, +23212, +23217) were used for this analysis.

Next, we assessed correlation of *SLC6A4* methylation status among average PCGI, PNCGI and NPNCGI using the Spearman correlation analysis. Methylation level of PCGIs and PNCGIs were positively correlated (R = 0.22, *p* = 0.005), while inverse correlation in methylation level was found between PCGIs and NPNCGIs (R = −0.59, *p*<0.0001). There was no significant correlation among methylation status of PNCGI and NPNCGI (Fig. 3).

### Methylation status of SLC6A4 in FD patients

**Figure 4:**
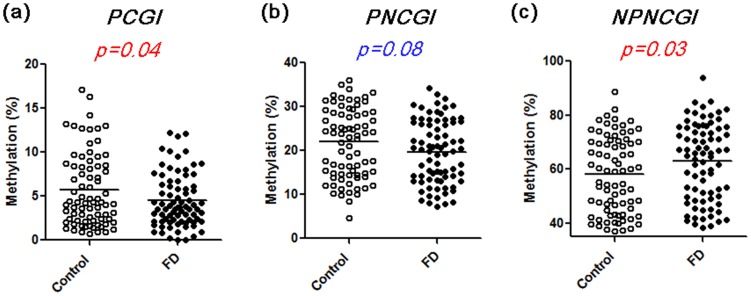
Methylation status of PCGI (a), PNCGI (b) and NPNCGI (c) of the *SLC6A4* in the gastric mucosa among FD and controls. Horizontal bars represent mean methylation level (%).

**Figure 5:**
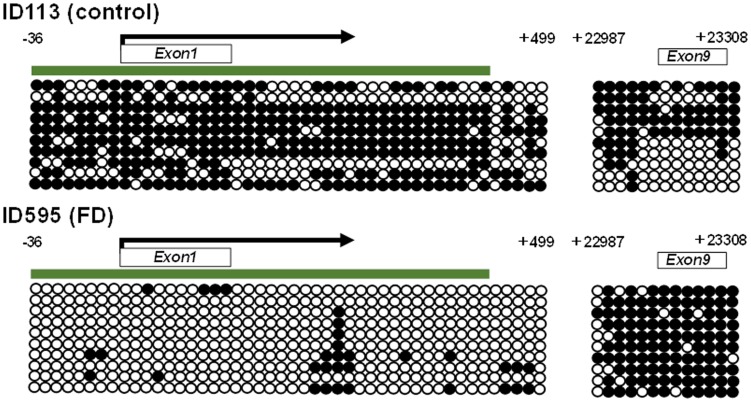
Representative bisulfite cloning sequencing of the *SLC6A4* PCGI, PNCGI (-36∼+499) and NPNCGI (+22987∼+23308) in FD and control. Open and filled circles represent unmethylated and methylated CpG sites, respectively. Transcriptional start site is by indicated by an arrow on the top. CpG islands (CGI) are indicated as green bar on the top.

**Figure 6:**
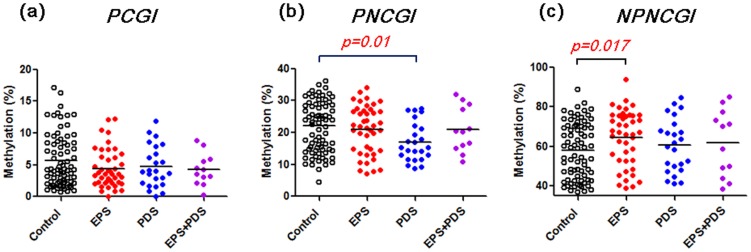
Methylation status of PCGI (a), PNCGI (b) and NPNCGI (c) of the *SLC6A4* in the gastric mucosa and subtypes of FD. Horizontal bars represent mean methylation level (%).

Methylation status of *SLC6A4* in FD and controls are shown in Fig. 4. In overall, we found that average methylation level of PCGI was significantly lower in FD compared to control subjects (4.5+/−0.3% vs. 5.7+/−0.5%, *p* = 0.04). Methylation level of PNCGI was also tended to be lower in FD compared to controls (19.7+/−0.8% vs. 22.0+/−1.0%, *p* = 0.08). On the other hand, methylation level of NPNCGI was significantly higher in FD compared to control subjects (62.9+/−1.6% vs. 58.0+/−1.6%, *p* = 0.03). Bisulfite cloning sequencing confirmed that a FD patient with lower methylation level in PCGI and PNCGI presents wide spread of lower methylation of surrounding CpG sites, analyzed by bisulfite pyrosequencing. The same patient also presented higher methylation level in CpG sites around the NPNCGI (Fig. 5). We next evaluated whether methylation status of *SLC6A4* would be associated with different subtypes of FD according to Rome III. Lower methylation level in PNCGIs was highlighted in the patients with PDS (*p* = 0.01), while higher methylation level in NPNCGIs seemed to be prominent in the patients with EPS (*p* = 0.017) (Fig. 6).

### Correlation of SLC6A4 methylation and gene expression

For 38 FD patients, correlation of *SLC6A4* methylation and gene expression was evaluated.

**Figure 7:**
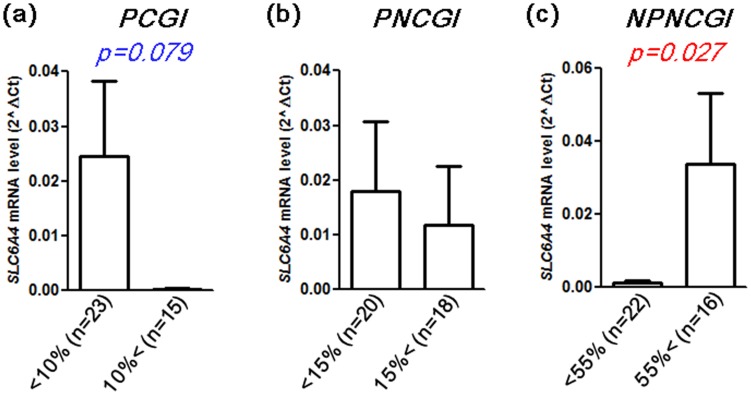
Methylation status of PCGI (a), PNCGI (b) and NPNCGI (c) of the *SLC6A4* in relation to mRNA expression. Data were expressed as mean +/−SEM.

Methylation levels of *SCL6A4* gene in the 38 FD patients had approximately Gaussian distribution across all the sites, with overrepresentation of higher methylation cases. On the basis of this, we set cutoff values of 10% in PCGI, 15% in PNCGI, 55% in NPNCGI, respectively. In NPNCGI, methylation level was positively correlated with *SLC6A4* mRNA level (*p* = 0.027). In CGI and PNCGI, the association seemed to be opposite, however, only weak association was found between higher PCGI methylation and lower *SLC6A4* mRNA level (*p* = 0.078). As for the PNCGIs, the association was not significant (Fig. 7).

## Discussion

Because of the potential role of SLC6A4 in FD, DNA methylation status of *SLC6A4* gene was examined in the gastric mucosa of FD patients. Methylation status of *SLC6A4* PCGI has been reported to have influence on serotonin transporter mRNA levels [17]. Recent research has also gradually revealed that change in methylation patterns across the gene (so-called intragenic or gene body methylation) may have a role in several pathologic state [19]. Thus, to obtain detailed information about methylation status, we also evaluated the methylation levels of *SLC6A4* throughout the gene, including promoter PCGI and NPNCGI. In FD, methylation status of *SLC6A4* in the gastric mucosa was lower in PCGI and higher in NPNCGI. PNCGI presented similar trend as PCGI. DNA methylation is involved in many biological phenomena [20], [21] and series of researches have demonstrated that change in DNA mehtylation status is involved in various pathological states such as aging, inflammation and cancer [22]. Recent studies have also suggested the epigenetic involvement in psychiatric disorders [18]. 5-HTT has an important role in the brain-gut communication and functional gastrointestinal disorders [13]. Recent result suggested the influence of DNA methylation in *SCL6A4* in the promoter region on the 5-HTT mRNA levels [17]. The current result indicates that epigenetic alteration of *SCL6A4*, characterized as low methylation in PCGI and high methylation in NPNCGI may have a pathogenic role in FD patients.

Regarding DNA methylation and mRNA expression, in general, higher methylation level of PCGI leads to lower mRNA level (transcriptional silencing) [22]. In 38 FD patients, weak correlation was found between higher PCGI methylation and lower *SLC6A4* mRNA level. A positive correlation between NPNCGI methylation and higher *SLC6A4* mRNA level was also observed. Therefore, change in methylation status of *SCL6A4* in FD patients, characterized as low in PCGI and high in NPNCGI is associated with higher mRNA expression of SCL6A4, leading to higher reuptake of serotonin in the presynaptic nerve terminals. In general, methylation level increase by the distance from the transcription start site [19]. To confirm this issue, we averaged methylation levels of *SLC6A4* I in all sites and assessed correlation methylation status among PCGI, PNCGI and NPNCGI. Our data demonstrated that *SLC6A4* methylation level in the gastric mucosa also increase by the distance from the transcription start site. The positive correlation in methylation levels among PCGIs and PNCGIs was observed. On the other hand, inverse correlation in methylation level was found between PCGIs and NPNCGIs. Our result suggest that methylation in the *SCL6A4* gene in the gastric mucosa also provides similar pattern as shown in common genes, characterized as low methylation around the promoter and gradual increase by the distance from the transcription start site. Since methylation status of PCGI and NPNCGI was inversely correlated, positive correlation between NPNCGI methylation and *SLC6A4* mRNA level seems to be reasonable. On the other hand, DNA methylation in NPNCGI has been also thought to have influence on gene expression through transcriptional factor binding [19]. The NPNCGI in the *SLC6A4*, analyzed in this study have numerous transcriptional factor binding sites but the role of these transcriptional factors in FD seemed to be unclear (https://genome.ucsc.edu/FAQ/FAQblat.html). Therefore whether the methylation change of *SLC6A4* NPNCGI would be a cause or consequence of other regulatory mechanisms needs to be investigated. SERT signaling plays an important role for brain-gut communication and functional gastrointestinal disorders [13]. Several reports have demonstrated that genetic polymorphism *SLC6A4*, affecting 5-HTT expression level is associated with susceptibility to functional gastrointestinal disorders [14]–[16]. Together with the comparison of methylation status among FD and control, our result suggests that epigenetic alteration of *SLC6A4*, leading to higher mRNA level may be associated with subset of FD.

We have also shown that lower methylation level in PNCGIs, higher methylation level in NPNCGIs was especially associated with PDS and EPS, respectively. These associations indicate that different FD subgroups have different molecular backgrounds. FD is so diverse in its symptom, clinical course and response to treatment and this heterogeneity is explained by complex pathophysiologic mechanisms, which is not conclusive. Our result suggest that low methylation level in PNCGIs and high methylation level in NPNCGIs would be useful molecular marker in PDS and EPS, respectively. In addition, association between methylation level of *SLC6A4* with specific subtypes of FD suggest that different epigenetic status of *SLC6A4* may one of the explanation of heterogeneity of FD, which needs to be mechanistically clarified. Our data demonstrated that, although significant, differences of SLC6A4 methylation status seen in this study are mostly small with large overlap between cases and controls, suggesting the pathogenic role of *SLC6A4* methylation status only in a subset of FD patients. The small sample size and marginal statistical difference could not obtain strong statistical power, which should be addressed as the limitation of our study. Since this is the first reporting the potential role of epigenetics in the development of FD, further well designed study will be needed to confirm our finding.

## Supporting Information

Figure S1 **Reproducibility of pyrosequencing determined for positions +734 and +736.**
(TIF)

Table S1 **Primer sequences used in bisulfite pyrosequencing.**
(DOCX)

Table S2 **Primer sequences used in bisulfite cloning sequencing.**
(DOCX)

Table S3 **Primer sequences used in real time PCR.**
(DOCX)
