# Supplementary material for: Change in DNA Methylation Patterns of SLC6A4 Gene in the Gastric Mucosa in Functional Dyspepsia
Source: PLoS One. 2014 Aug 22;9(8):e105565. doi: 10.1371/journal.pone.0105565 (PMC4141787; doi:10.1371/journal.pone.0105565)
Supplement: Table S1 — Primer sequences used in bisulfite pyrosequencing. (DOCX) [file pone.0105565.s002.docx]

| **Supplementary Table 1. Primer sequences used in bisulfite pyrosequencing** | | | | |  |  |
| --- | --- | --- | --- | --- | --- | --- |
| Assay name | Forward primer (1st and 2nd step PCR) | Reverse primer (1st step PCR) | Reverse primer (2nd step PCR) | Sequencing primer |  |  |
|  | sequence | sequence | sequence | sequence |  |  |
| *SLC6A4* PCGI | AGAGATTAGATTATGTGAGGGTT | AAAAAAAAAACCAACCCACCTACCAA | U-AAAAAAAAAACCAACCCACCTACCAA | GAGGGTTAGAGGGTATAAAT |  |  |
| *SLC6A4* NPCGI | AGAGGGTTTTGAGTAAAGTGT | AAACATTTATATCAACCAAAACTCTCC | U-AAACATTTATATCAACCAAAACTCTCC | ATGTTTGATTTTTATTTTGTTGTTA |  |  |
| *SLC6A4* NPNCGI | TGGGTTTTTTTTTTTAGAGATGTT | TTCATTCCTCATCTCAACCATATA | U-TTCATTCCTCATCTCAACCATATA | TAGAGATGTTTTGGTGATTA |  |  |
| U =biotin labeled universal primer tag: 5'-biotin-GGGACACCGCTGATCGTTTA | | |  |  |  |  |
