# Supplementary material for: Change in DNA Methylation Patterns of SLC6A4 Gene in the Gastric Mucosa in Functional Dyspepsia
Source: PLoS One. 2014 Aug 22;9(8):e105565. doi: 10.1371/journal.pone.0105565 (PMC4141787; doi:10.1371/journal.pone.0105565)
Supplement: Table S2 — Primer sequences used in bisulfite cloning sequencing. (DOCX) [file pone.0105565.s003.docx]

| **Supplementary Table 2.** Primer sequences used in bisulfite cloning sequencing | | |
| --- | --- | --- |
| Assay name | Forward primer | Reverse primer |
|  | sequence | sequence |
| *SLC6A4* PCGI-PNCGI | AGAGATTAGATTATGTGAGGGTT | AAACATTTATATCAACCAAAACTCTCC |
| *SLC6A4* NPNCGI | TGTGTGGTGGTTATGGTAGTTAGA | CCACCATACCCAACCTTCTTT |
