# Supplementary material for: Change in DNA Methylation Patterns of SLC6A4 Gene in the Gastric Mucosa in Functional Dyspepsia
Source: PLoS One. 2014 Aug 22;9(8):e105565. doi: 10.1371/journal.pone.0105565 (PMC4141787; doi:10.1371/journal.pone.0105565)
Supplement: Table S3 — Primer sequences used in real time PCR. (DOCX) [file pone.0105565.s004.docx]

| **Supplementary Table 3.** Primer sequences used in bisulfite cloning sequencing | | |
| --- | --- | --- |
| Assay name | Forward primer | Reverse primer |
|  | sequence | sequence |
| 5HT RT-PCR | TCCCGCTCTTTTACATGGAG | GATGCAGATGGCATAACCAA |
| GAPDH | AGCCACATCGCTCAGACAC | GTTAAAAGCAGCCCTGGTGA |
|  |  |  |
